# Differential associations between experiences of contraceptive care and subsequent contraceptive access and preferences among family planning patients by racial and ethnic identity: Evidence from Arizona, Iowa, and Wisconsin

**DOI:** 10.1371/journal.pone.0312111

**Published:** 2024-10-11

**Authors:** Megan L. Kavanaugh, Madeleine Haas, Ayana Douglas-Hall

**Affiliations:** Research Division, Guttmacher Institute, New York, NY, United States of America; National Institute of Public Health: Instituto Nacional de Salud Publica, MEXICO

## Abstract

While many frameworks exist for building person-centered and equitable systems of contraceptive care, evidence indicates that the reality of patients’ experiences of care is often not in alignment with these ideals. Historical and current contexts of racism in the healthcare system contribute to negative perceptions and experiences of care, as well as reduced care-seeking behavior, for those who identify as Black, Indigenous, and people of color (BIPOC). Our objective in this analysis is to examine whether people’s past experiences of contraceptive care are a driver of subsequent barriers to contraceptive access, and whether this relationship differs across racial and ethnic identity. We draw on panel data from five waves of surveys collected between 2018–2023 among patients ages fifteen and older seeking family planning care at sites that receive public funding for these services in Arizona, Iowa, and Wisconsin. Overall and stratified by race/ethnicity, we examine cross-sectional and longitudinal associations between patients’ experiences of high-quality, person-centered contraceptive care and three contraceptive access outcomes: use of preferred contraception, satisfaction with contraceptive method, and experience of no barriers to accessing preferred contraception. We find longitudinal associations between patients experiencing higher-quality, more person-centered contraceptive care and subsequent satisfaction with contraceptive methods. Among non-Hispanic white-identifying patients, we find similar associations between shifting to higher-quality contraceptive care and use of preferred contraception, but we find no statistical relationship between experiencing higher-quality care and subsequent contraceptive outcomes for patients who identify as Black, Indigenous, or Person of Color (BIPOC). Highlighting the dissonance between clinical guidance for quality contraceptive care and patient experiences of care, and especially whether similar experiences across racial and ethnic identity lead to differential outcomes, is a crucial step toward bringing contraceptive care systems into alignment with principles of sexual and reproductive health equity.

## Introduction

Reproductive autonomy, or the ability to be fully empowered in reproductive decisions and to access reproductive health services without interference or coercion, requires access to desired contraceptive care [1, 2]. Contraceptive care should be high quality, equitable, and accessible to all those who need and want it, and it should be built from a framework that considers an individual’s community, family, health systems, and socio-economic contexts [3, 4]. Person-centered care, or care that prioritizes a person’s lived experiences, desires, and reproductive autonomy, is identified as an essential domain of health care quality by the National Academies of Medicine [5]. Healthcare systems realize sexual and reproductive health equity when they are aligned with principles of person-centered care and are built to ensure that all people, regardless of age, race, sex, gender and other intersectional identities, have what they need to achieve their highest level of sexual and reproductive health and well-being [6].

While many frameworks exist for imagining these person-centered and equitable systems of contraceptive care, evidence indicates that the reality of patients’ experiences of care is often not in alignment with these ideals. Historical and current contexts of racism, reproductive coercion, and abuse of individuals who identify as Black, Indigenous, and people of color (BIPOC) within the healthcare system contribute to negative perceptions and experiences of care, as well as reduced care-seeking behavior [2, 7–10]. Evidence indicates that patient characteristics, particularly race and class, are associated with the type and quality of family planning services received; providers disproportionately recommend long-acting reversible contraception to low-income women of color, regardless of their contraceptive preferences [11], and low-income Black and Latina women report higher levels of discrimination within contraceptive counseling discussions than their middle-income, white counterparts [7]. While around 65% of sexually active women aged 18–49 are using contraceptives, only about 40% of them report having received person-centered contraceptive care, and only 30% report having all the information they needed to choose a method [12]. Uninsured women and those identifying as non-Hispanic Black, Spanish-speaking Hispanic, and non-Hispanic other or multiple racial identities report lower levels of person-centered contraceptive care than their insured and non-Hispanic white counterparts, respectively [12, 13].

In addition to differential experiences of contraceptive care, inequities in access to this care, including realization of contraceptive preferences, persist. Nationally, 23% of contraceptive method users and 39% of people not using a method would prefer to use a different method if cost was not an issue [14]; experiences of person-centered contraceptive counseling reduce the odds of reporting unfulfilled contraceptive preferences [14]. More broadly, 25% of contraceptive method users aren’t using their preferred method; reasons for this vary by ethnicity, income, and age [12]. Misalignment of contraceptive preferences and actual contraceptive use is associated with discontinued method use and pregnancy [15].

Highlighting the dissonance between clinical guidance for quality contraceptive care and patient experiences of care is a crucial step toward bringing contraceptive care systems into alignment with the principles of sexual and reproductive health equity. This is especially the case with regards to these systems valuing and supporting everyone justly and fairly to attain their highest level of sexual and reproductive health [6]. To move in this direction, we need to understand whether people’s past experiences of care are a driver of subsequent barriers to access, and whether this relationship differs across axes of identity. Much of the existing literature examining the interplay between these two research domains—contraceptive care experiences and realization of contraceptive preferences—is comprised of qualitative studies and cross-sectional analyses of survey data [16, 17]. Our analysis draws on a unique panel dataset of family planning patients to examine whether experiences of contraceptive care are associated with subsequent realization of contraceptive preferences and, given broad evidence on poorer experiences of contraceptive care among BIPOC individuals compared to those identifying as white, whether this relationship differs according to racial and ethnic identity. We hypothesized that more positive contraceptive care experiences would lead to fewer barriers to attaining contraceptive care and greater use of preferred contraceptive methods over time and that this pathway would differ according to racial and ethnic identity.

## Methods

### Data

For this analysis, we drew on longitudinal survey data collected under a larger research initiative called the Reproductive Health Impact Study (RHIS), a multistate, multiyear effort to document the impact of policy changes on the publicly funded family planning health care system and on those who rely on it for care [18]. We drew on panel data from five waves of the RHIS Family Planning Clinic Patient Surveys which, at baseline, are representative of patients ages fifteen and older seeking family planning care at sites that receive public funding for these services in Arizona (AZ), Iowa (IA), and Wisconsin (WI). The nonpartisan and objective research organization NORC at the University of Chicago (formerly called the National Opinion Research Center) managed all electronic data collection for these surveys. More detail on sampling and data collection protocols for these baseline surveys has been published elsewhere [19].

All study participants initially provided verbal consent to receiving information about the study at baseline. For all web-based surveys, the first screen presented prior to entering the electronic survey included a statement of informed consent. In order to officially proceed to initiate the survey, respondents pressed a submit button. This passive consent is the field standard for most web surveys for Federal and Non-Federal patients [20]. Participants of all ages in the study, including minors ages 15–17, followed this process. Federal guidelines allow for minor adolescents who receive family planning services to do so without the consent of a parent; thus, the IRB deemed minors obtaining family planning services in this study as capable of consenting to complete a questionnaire.

Seventy-one percent of the 6,074 respondents who completed the baseline surveys in these three states agreed to be contacted for subsequent, biannual follow-up surveys during the following 24 months. NORC sent a survey link in the mail and/or via email to those who opted to participate in these longitudinal surveys. NORC offered respondents incentives, starting at $15 total at baseline and increasing by $5 with each follow-up survey. Incentives were paid mainly through Amazon gift codes after survey completion, however, NORC sent an initial $5 cash pre-incentive to those invited to participate by mail for each follow-up survey. All survey instruments included questions about contraceptive use and satisfaction, sexual and reproductive health care use and access, and demographic information, among other foci. NORC formatted the follow-up surveys to be accessible online on the secure Voxco platform; respondents took approximately 10–15 minutes to complete.

This analysis includes data from the baseline through 24-month follow-up surveys. Iowa data cover the period of May 2018 through February 2021, Arizona data cover the period of May 2019 through January 2022, and Wisconsin data cover the period from June 2020 to August 2023. Data are weighted to reflect the universe of patients served at publicly funded family planning centers in 2018 (Iowa and Arizona) or in 2020 (Wisconsin) [21].

Our and our data management partner’s Institutional Review Boards (DHHS identifier IRB00002197) approved all study protocols for the Family Planning Clinic Patient Surveys.

### Measures

Our analytic sample comprises pooled data from respondents across the three states. Given our overall research question to understand the sequential relationship between contraceptive care experiences and subsequent contraceptive access and preferences, we limit eligibility for our analysis to baseline survey respondents who received contraceptive services at least once in the year prior to baseline survey completion. We conduct analyses with both this full baseline sample and with a longitudinal sample that further limits eligibility to respondents who completed at least one follow-up survey. The longitudinal sample represents an unbalanced panel, with 53% completing all five rounds of data collection and the rest participating in two to four rounds.

We considered contraceptive care experiences as our key independent variable. To reflect quality of contraceptive care experiences, we used the Person-Centered Contraceptive Counseling (PCCC) metric, a validated 4-item measure in which respondents rate the healthcare provider they saw most recently for birth control-related services with regards to four key aspects of care [22]. These include: “Respecting me as a person,” “Letting me say what mattered to me about my birth control method,” “Taking my preferences about my birth control seriously,” and “Giving me enough information to make the best decision about my birth control method.” Each of the items is rated on a 5-point likert scale from “Poor” to “Excellent”, and the final PCCC score is calculated as the percentage of respondents who gave a top score (“Excellent”) on all four items. We operationalized the final measure in this way to align with published guidance on using it for analytic purposes and to account for the skewedness toward positive scores found in many patient satisfaction and experience measures [23, 24].

We examined three key outcomes representing access to contraception in this analysis, including use of preferred contraception, satisfaction with contraceptive methods used, and experience of no barriers to realizing contraceptive preferences. We determined use of a preferred method among all respondents—both contraceptive users and nonusers—at all survey timepoints (except for IA baseline) by responses to the question “*If you could use any birth control method you wanted*, *what method(s) would you use*?,” with options including “I’m using the method that I want to use” followed by a list of contraceptive methods and an open-ended option. We consider both users and nonusers who indicated "*I am using the method that I want to use*" to be using their preferred contraception, including not using a method if that is the stated preference. To assess satisfaction with current method(s), respondents who reported current contraceptive method use in the three months prior to each of the survey time points rated how satisfied they were with their current birth control method on a 5-point likert scale from 1 (Very satisfied) to 5 (Very unsatisfied). We recoded responses into a binary variable, grouping very and somewhat satisfied into the satisfied category and the remaining three categories into not satisfied. To assess experiences of no barriers to realizing contraceptive preferences, all respondents indicated if they had “*delayed or had trouble getting the birth control method [they] wanted for any reason*” in the last 12 months in the baseline survey. This question was asked only of contraceptive users in the AZ and WI 6-month follow-up and in all other subsequent follow-up surveys across states at 12, 18, and 24 months. We considered responses of “no” to represent experiencing no barriers to preferred contraception.

We included several variables describing the study population at baseline including age, household income as a percentage of the federal poverty level, ethnicity, race, sexual and gender minority status, educational attainment, employment level, relationship status, nativity to the U.S., and health insurance coverage. Respondents reported their ethnicity as identifying as either “Hispanic, Latina or Latinx” or “none of these,” with "Hispanic" primarily being associated with having a Spanish-speaking origin, whereas "Latino" and "Latinx" being associated with broader categories encompassing people of Latin American descent, and "Latinx" being a gender-neutral option. We determined racial identity of respondents based on responses to the baseline survey question “*which one or more of the following best describes your race*?” with options to choose one or more of the following: Black or African American, white, Asian or Asian American, Native American, Alaska Native, or American Indian, Native Hawaiian or Pacific Islander, and an open-ended box for respondents to write-in other racial identities. During analysis we created an additional “multiracial” category to capture all respondents who reported more than one racial identity. For analytic purposes, due to smaller percentages of some racial identities in the sample, we also created two additional variables that condensed racial identity into either four categories (Black/African American; white; Asian or Asian American or Pacific Islander (AAPI), Native, or Other; and Multiracial) or two categories (BIPOC vs. Non-Hispanic white). We determined this latter categorization of the binary BIPOC variable based on responses to both the ethnicity and racial identity questions.

### Analytic strategy

We first conducted descriptive analyses of our sample. We then examined cross-sectional associations between our key independent variable and each of our three outcome variables via logistic regression, adjusting for respondent characteristics, all drawing on baseline data from across the three states. We then examined changes in our key variables over time, including chi-square tests to determine whether each of these changed significantly over the full study period. We conceptualized changes in the variables as being “consistently yes” (respondents who indicated experiencing the outcome at every time point for which we had data, e.g., respondents who reported experiencing high quality contraceptive care at every time point), “consistently no” (respondents who indicated not experiencing the outcome at every time point for which we had data, e.g., respondents who did not use their preferred method at any time point), “any change from yes to no” (respondents who indicated “yes” on an outcome in an earlier survey and “no” to the same outcome in any later survey for which we had data, e.g., respondents who were satisfied with their method at baseline, but were dissatisfied with their method at any subsequent time point) and “any change from no to maintenance of yes” (respondents who indicated “no” on an outcome in an earlier survey and “yes” to the same outcome in every subsequent survey for which we had data, e.g., respondents who had delays/trouble getting preferred method at baseline, but no delays/trouble at any subsequent time point).

Finally, we estimated conditional logistic regression models that controlled for observed (measured within datasets) and unobserved (not measured) non-changing characteristics within individuals to examine fixed effects of the experience of contraceptive care (measured by the PCCC) on the three outcomes (use of preferred contraceptive method, satisfaction with contraceptive method used, and no barriers to accessing preferred contraception). In each model, we adjusted for key time-varying sociodemographic variables that have been linked to contraceptive access in other research: sexual and gender minority status, employment, relationship status, and health insurance coverage. We ran each of the three models overall and, given baseline cross-sectional differences in two of the outcomes by racial/ethnic identity (use of preferred method and satisfaction with method used), we also ran each model stratified by the binary race/ethnicity identity variable. We use the binary version of the race/ethnicity identity variable in all multivariable models given small numbers of respondents identifying as a race/ethnic group besides non-Hispanic white. We lagged the PCCC measure in our final model examining the relationship between the receipt of quality contraceptive care and no barriers to accessing preferred contraception to ensure that the timeframe covered by this key independent variable preceded that of the outcome. For all models, we present adjusted odds ratios, 95% confidence intervals, and p values; we highlight findings in which associations between predictors and outcomes are significant at the p<0.05 level. We based all analyses on weighted data and we used the svy command in Stata 18.0 to account for the complex sampling designs.

## Findings

We present characteristics of the analytic sample of family planning patients in Arizona, Iowa, and Wisconsin who reported receiving recent contraceptive care during baseline surveys, as well as characteristics of the narrower sample who also completed at least one follow-up survey, in Table 1. For both these samples, the majority of these patients were ages 20–29, had incomes at 200% or more of the federal poverty level, had at least some college or associate degree, were employed for wages, and had some form of health insurance. A majority also identified as non-Hispanic, white, having been born in the U.S., as straight, and cisgender, while about half reported not living with a romantic partner nor being married. The majority of the sample indicated using some form of contraception (90%, unweighted N = 2797). At baseline, 66% (unweighted N = 2046) indicated that their recent contraceptive care had been person-centered. When examining racial differences in experiences of quality care, patients identifying as white reported higher baseline levels on each of the four domains of the PCCC (between 3–7 percentage points) than those identifying as BIPOC (not shown).

**Table 1:** Characteristics of Arizona, Iowa, and Wisconsin family planning patients ages 15+ in analytic samples, baseline (2018–2020) and longitudinal (2018–2023).

	Baseline sample, pooled across states		Longitudinal sample, pooled across states	
	n	Weighted %	n	Weighted %
**Total**	3076	100	1801	100
**Age (years)**				
15–19	610	18.3	271	13.6
20–29	1,758	54.0	1,089	58.1
30–39	522	21.2	349	23.3
40+	186	6.4	92	5.0
**Income level**				
<100% FPL	1,113	25.1	593	23.0
100–199% FPL	906	21.7	555	20.9
200%+ FPL	1,057	53.3	653	56.1
**Hispanic, Latina or Latinx**				
No	2,284	65.2	1,323	64.3
Yes	756	34.8	469	35.7
**Race**				
White	2,017	78.1	1,209	77.0
Black or African American	443	10.7	229	10.5
Asian or Asian American	73	2.4	60	3.3
Native American, Alaska Native, or American Indian	41	2.2	25	2.7
Native Hawaiian or Pacific Islander	7	0.3	3	0.1
Multiracial	227	6.2	118	6.3
Other	4	0.1	3	0.1
**Sexual orientation**				
Straight	2,431	84.5	1,371	80.9
Bisexual, queer, pansexual, or other^a^	566	15.1	386	18.5
Lesbian, gay	14	0.4	8	0.6
**Gender identity**				
Cisgender/Woman	3,032	99.1	1,774	98.9
Gender non-conforming/Other	23	0.5	19	0.8
Transgender	15	0.3	7	0.3
**Educational attainment**				
Less than high school	319	11.0	104	6.4
HS graduate or GED recipient	884	27.7	490	24.7
Some college or associate degree	1,204	37.2	750	40.3
College graduate or more	648	24.1	450	28.5
**Employment level**				
Employed for wages	1,735	59.7	1,040	61.8
Self-employed	155	5.2	90	5.9
A student	751	21.3	404	18.2
Out of work for 1 year or more	61	1.8	41	2.0
Out of work for less than 1 year	155	4.7	107	6.1
A homemaker	109	5.0	57	4.5
Retired	2	0.2	1	0.3
Unable to work	64	2.0	36	1.2
**Relationship status**				
Not living with romantic partner, never married	1,724	50.8	996	49.3
Living with romantic partner, not married	769	26.2	453	25.4
Married	349	15.7	223	18.1
Formerly married, not cohabitating	199	7.2	115	7.3
**Nativity**				
Born in the United States (including territories)	2,805	88.6	1,629	88.9
Born outside of the United States	260	11.4	169	11.1
**State**				
Arizona	963	51.6	613	54.4
Iowa	1,254	25.9	828	30.4
Wisconsin	859	22.5	360	15.2
**Health insurance coverage**				
Private insurance	1,229	44.1	732	45.8
Public insurance	1,102	29.9	646	28.9
Other insurance	137	4.8	78	4.0
No insurance	509	21.3	303	21.2
**Contraceptive Use**				
Yes	2,797	89.6	1,613	88.0
No	276	10.4	185	12.0
**Experience of high-quality contraceptive care** ^d^				
Yes (Excellent)	2,046	66.2	813	66.5
No	996	33.8	385	33.5

Note: Eligibility for the analytic samples was limited to baseline survey respondents who received contraceptive services at least once in the year prior to baseline survey completion and, for the longitudinal sample, to those who also completed at least one follow-up survey.

^a^ Queer was a response option given in Wisconsin. The Arizona and Iowa surveys did not include queer as a response option, however, some participants wrote in this response.

^b^ Private insurance includes employer‐based plans and plans purchased on the marketplace or exchange. Public insurance options include Medicaid, Medicare, Tricare, Indian Health Service.

^c^ Respondents who received contraceptive services were asked to rate the quality of this care received. This item is a composite of four measures including: "Respecting me as a person", "Letting me say what mattered to me about my birth control method", "Taking my preferences about my birth control seriously", "Giving me enough information to make the best decision about my birth control method". Respondents were asked to score each measure on a 5-point Likert scale. Respondents who scored their care as ’Excellent’ on all four measures are coded as having received quality contraceptive care.

FPL, federal poverty level; GED, General Educational Development

We present cross-sectional associations between the key independent variables of contraceptive care experiences, racial identity (four category variable), ethnicity, and three outcomes representing contraceptive access in Table 2. Adjusting for respondent demographics, at baseline those who reported receiving recent person-centered contraceptive care had higher adjusted odds of using a preferred method of contraception (aOR = 1.9, CI = 1.3–2.6), satisfaction with their method (aOR = 1.7, CI = 1.2–2.4), and experiencing no delays or trouble in realizing their contraceptive preferences (aOR = 2.0, CI = 1.5–2.7).

**Table 2:** Cross-sectional associations between contraceptive care experiences and racial/ethnic identity and three contraceptive access outcomes among Arizona, Iowa, and Wisconsin family planning patients at baseline (2018–2020).

	Use of preferred method(s)^b^					Satisfied with current method(s)^c^					No Delays/Trouble getting contraceptive care in past year^d^				
	n	aOR^e^	CI		p-value	n	aOR^e^	CI		p-value	n	aOR^e^	CI		p-value
**Experience of high-quality contraceptive care** ^a^	1,802	1.85	1.29 -	2.63	0.00	2,230	1.72	1.22 -	2.43	0.00	2,569	2.03	1.53 -	2.70	0.00
**Racial identity**															
White (ref.)		1.00					1.00					1.00			
Black or African American		1.01	0.58 -	1.76	0.97		0.72	0.38 -	1.34	0.29		1.08	0.59 -	2.00	0.79
AAPI, Native, or Other		2.71	1.02 -	7.17	0.04		1.58	0.65 -	3.82	0.30		0.73	0.42 -	1.28	0.27
Multiracial		1.04	0.56 -	1.93	0.91		1.68	0.94 -	2.99	0.08		0.93	0.45 -	1.90	0.83
**Ethnicity**															
Non-Hispanic (ref.)		1.00					1.00					1.00			
Hispanic		0.55	0.32 -	0.93	0.03		0.54	0.36 -	0.79	0.00		1.02	0.70 -	1.48	0.93

Note: Baseline sample includes baseline survey respondents who received contraceptive services at least once in the year prior to baseline survey completion.

^a^ Respondents who received contraceptive services were asked to rate the quality of this care received. This item is a composite of four measures including: "Respecting me as a person", "Letting me say what mattered to me about my birth control method", "Taking my preferences about my birth control seriously", "Giving me enough information to make the best decision about my birth control method". Respondents were asked to score each measure on a 5-point Likert scale. Respondents who scored their care as ’Excellent’ on all four measures are coded as having received quality contraceptive care.

^b^ This item was asked of all respondents in Arizona and Wisconsin at baseline. It was not asked of Iowa respondents at baseline.

^c^ This item was limited to current method users at baseline and in Arizona, Iowa, and Wisconsin.

^d^ This item was asked of all respondents in AZ, IA, and WI at baseline. However, it was not asked of AZ and WI respondents who reported no contraceptive use in follow-up surveys. This item was asked of all Iowa respondents in Iowa at the 6 months follow-up. It was not asked of IA respondents who reported no contraceptive use in 12- and 18-month follow-ups.

^e^ Multivariable models include time-invariant controls: sexual and gender minority status, employment, relationship status and health insurance coverage.

In adjusted models assessing the cross-sectional relationships between experiences of care and the outcomes of preferred contraceptive use and satisfaction with contraception, we found associations between respondents’ racial and ethnic identity and both of these outcomes. Specifically, when comparing to patients identifying as white, those identifying as AAPI, Native, or other race reported higher levels of preferred contraceptive use (aOR = 2.7, CI = 1–7.2) and those identifying as multiracial reported marginally higher levels of satisfaction with their current methods (aOR = 1.7, CI = 0.9–3). Compared to patients identifying as non-Hispanic, those identifying as Hispanic reported lower levels of preferred contraceptive use (aOR = 0.6, CI = 0.3–0.9) as well as lower levels of satisfaction with their current methods (aOR = 0.5, CI = 0.4–0.8).

Given the cross-sectional associations found between patients’ experiences of contraceptive care and the key outcomes, we present changes in these experiences and outcomes over time, overall and by racial and ethnic identity, in Table 3. Most patients reported receiving consistent person-centered contraceptive care (53%, unweighted N = 914), being consistently satisfied with their contraceptive method (65%, unweighted N = 1102), and consistently experiencing no delays or barriers to their preferred contraception (62%, unweighted N = 1131) across timepoints, with non-Hispanic white-identifying respondents having slightly higher levels of consistent use of preferred methods and satisfaction with methods than BIPOC respondents and BIPOC respondents having consistently higher levels of receiving high-quality care than non-Hispanic white respondents. Almost one-fifth of respondents (19%, unweighted N = 349) reported receiving consistently lower-quality, or non-person-centered care, and non-Hispanic white respondents reported slightly lower levels of this consistent lower quality care than BIPOC respondents (17%, unweighted N = 159; and 20%, unweighted N = 187, respectively). A smaller percentage of respondents indicated experiencing shifts towards more negative experiences and outcomes over time, with 17% (unweighted N = 327) indicating that they experienced a shift from high-quality to lower-quality care at any time over the study period, 42% (unweighted N = 694) indicating that they shifted from using to not using their preferred contraception, 18% (unweighted N = 333) indicating that they shifted from being satisfied to not being satisfied with their contraceptive method, and 28% (unweighted N = 476) indicating that they shifted from not experiencing barriers to their preferred contraception to experiencing these barriers over time. We observe that BIPOC respondents more commonly reported these negative shifts over time in use of preferred contraception and satisfaction with method use and less commonly reported shifts towards less high-quality care than did non-Hispanic white respondents. Less than 15% of respondents (unweighted N <229) indicated a positive shift from “no” to staying at “yes” on these experiences and outcomes over the study period. Slightly higher levels of BIPOC patients indicated this shift towards more positive outcomes of preferred use and satisfaction with methods than non-Hispanic white-identifying patients over time.

**Table 3:** Changes in experiences of contraceptive care and contraceptive access outcomes over study period among Arizona, Iowa, and Wisconsin family planning patients, longitudinal sample, 2018–2023.

	Consistently "Yes" across study period				Consistently "No" across study period				Any change from "Yes" to "No" across study period				Any change from "No" to maintenance of "Yes" across study period			
	n	Total %	Among NH white Rs %	Among BIPOC^e^ Rs %	n	Total %	Among NH white Rs %	Among BIPOC Rs %	n	Total %	Among NH white Rs %	Among BIPOC Rs %	n	Total %	Among NH white Rs %	Among BIPOC Rs %
Experience of high-quality contraceptive care^a^	914	53.2	50.7	55.9	349	19.1	17.4	20.0	327	16.5	19.3	14.1	199	11.2	12.6	10.0
**Outcomes of contraceptive access**																
Use of preferred methods^b^	756	37.3	43.3	31.9	122	7.0	7.2	6.7	694	41.8	38.6	45.1	229	14.0	10.9	16.2
Satisfaction with current methods^c^	1,102	64.6	70.3	58.5	86	5.0	3.6	6.3	333	18.2	15.7	20.9	196	12.2	10.4	14.2
No delays/trouble getting preferred contraception in past 6 months/year^d^	1,131	62.4	61.4	62.9	60	2.9	2.9	2.9	476	27.6	28.7	26.8	130	7.2	7.0	7.4

Note: Longitudinal sample includes baseline survey respondents who received contraceptive services at least once in the year prior to baseline survey completion and who also completed at least one follow-up survey. Respondents who consistently responded "yes" across the study period reported having a given outcome at every time point for which we have data, i.e., used their preferred method(s) at every time point. Respondents who had any change in report of a given outcome across the study period were categorized based on the directionality of that change, i.e., a respondent who had delays/trouble getting preferred contraception at baseline, then did not have delays/trouble at a later time point would be coded as "any change from ’yes’ to ’no’ across study period.

^a^ Respondents who received contraceptive services were asked to rate the quality of this care received. This item is a composite of four measures including: "Respecting me as a person", "Letting me say what mattered to me about my birth control method", "Taking my preferences about my birth control seriously", "Giving me enough information to make the best decision about my birth control method". Respondents were asked to score each measure on a 5-point Likert scale. Respondents who scored their care as ’Excellent’ on all four measures are coded as having received quality contraceptive care.

^b^ This item was asked of all respondents at all timepoints with the exception of respondents to the IA baseline survey. At baseline, respondents given the option to assert “I am not using the method that I want to use”. This option was not available in follow-up surveys. Some respondents who may have otherwise selected "prefer not to answer" or "don’t know" have been coded as "No" in follow-up surveys.

^c^ This item was limited to current method users at baseline and in all follow-up surveys in AZ, IA, and WI.

^d^ This item was asked of all respondents in AZ, IA, and WI at baseline. However, it was not asked of AZ and WI respondents who reported no contraceptive use in follow-up surveys. This item was asked of all Iowa respondents in Iowa at the 6 months follow-up. It was not asked of IA respondents who reported no contraceptive use in 12- and 18-month follow-ups.

^e^ Nine percent of the study population did not provide a racial identification. The categorization of the binary BIPOC variable is determined based on responses to racial identity and ethnicity questions.

We present associations between changes in experiences of person-centered contraceptive counseling and subsequent changes in realization of contraceptive preferences, satisfaction with contraception, and no barriers to realizing contraceptive preferences as adjusted odds ratios in Table 4. After adjusting for all time-invariant sociodemographic characteristics as well as the time-varying variables of sexual and gender minority status, employment, relationship status, and insurance coverage status, those who changed from receiving less to more person-centered care had marginally increased adjusted odds of being satisfied with their contraceptive method (aOR = 1.9, CI = 1–3.7). Among the full study population, we detected no significant association between changes in experiences of contraceptive care and subsequent reports of realization of preferred method use or no delays or trouble realizing contraceptive preferences.

**Table 4:** Associations between contraceptive care experiences and subsequent contraceptive access outcomes, assessed via fixed effects conditional logistic regression models and stratified by racial/ethnic identity, among Arizona, Iowa, and Wisconsin family planning patients, longitudinal sample, 2018–2023.

	Use of preferred method(s)^c^ (n = 1,295)				Satisfied with current method(s)^d^ (n = 747)				No Delays/Trouble getting contraceptive care in past 6 months/year^e^ (n = 931)			
Experience of high-quality contraceptive care^a^	aOR^f^	CI		p-value	aOR^f^	CI		p-value	aOR^f^	CI		p-value
Overall												
No	1.00				1.00				1.00			
Yes	1.42	0.93 -	2.15	0.10	1.90	0.99 -	3.68	0.06	1.36	0.80 -	2.31	0.26
Among NH white Rs^b^												
No	1.00				1.00				1.00			
Yes	2.43	1.21 -	4.91	0.01	2.13	0.89 -	5.11	0.09	1.38	0.64 -	2.98	0.41
Among BIPOC Rs^b^												
No	1.00				1.00				1.00			
Yes	0.81	0.47 -	1.38	0.43	1.84	0.67 -	5.07	0.23	1.30	0.53 -	3.21	0.56

Note: Records ("n") represent the number of contributing data points from all respondents across the study period. Longitudinal sample includes baseline survey respondents who received contraceptive services at least once in the year prior to baseline survey completion and who also completed at least one follow-up survey.

^a^ Respondents who received contraceptive services were asked to rate the quality of this care received. This item is a composite of four measures including: "Respecting me as a person", "Letting me say what mattered to me about my birth control method", "Taking my preferences about my birth control seriously", "Giving me enough information to make the best decision about my birth control method". Respondents were asked to score each measure on a 5-point Likert scale. Respondents who scored their care as ’Excellent’ on all four measures are coded as having received quality contraceptive care.

^b^ Nine percent of the study population did not provide a racial identification. The categorization of the binary BIPOC variable is determined based on responses to racial identity and ethnicity questions.

^c^ This item was asked of all respondents at all timepoints with the exception of respondents to the IA baseline survey. At baseline, respondents given the option to assert “I am not using the method that I want to use”. This option was not available in follow-up surveys. Some respondents who may have otherwise selected "prefer not to answer" or "don’t know" have been coded as "No" in follow-up surveys.

^d^ This item was limited to current method users at baseline and in all follow-up surveys in AZ, IA, and WI.

^e^ This item was asked of all respondents in AZ, IA, and WI at baseline. However, it was not asked of AZ and WI respondents who reported no contraceptive use in follow-up surveys. This item was asked of all Iowa respondents in Iowa at the 6 months follow-up. It was not asked of IA respondents who reported no contraceptive use in 12- and 18-month follow-ups.

^f^ Multivariable models include time-invariant controls: sexual and gender minority status, employment, relationship status and health insurance coverage.

When stratifying the sample by race/ethnicity, among respondents who identify as non-Hispanic white, compared to those who did not experience a change in the quality of contraceptive care received over time, we find those who reported experiencing higher-quality contraceptive care over time had higher odds of subsequently using their preferred contraceptive method (aOR = 2.4, CI = 1.2–4.9) and marginally higher odds of being satisfied with their contraceptive method (aOR = 2.1, CI = 0.9–5.1). On the other hand, among respondents who identified as BIPOC, we find no statistically significant associations between changes in care experiences and these outcomes, although the pattern is in the same direction as with those who identify as non-Hispanic white.

## Discussion

Individuals’ experiences of contraceptive care influence how, and whether, they are able to access their preferred contraception and, thus, realize this key aspect of reproductive autonomy. This study confirms that publicly supported family planning patients in Arizona, Iowa, and Wisconsin demonstrate similar cross-sectional relationships between contraceptive care experiences and access to contraception to what has been identified for broader and other populations in the literature. Our findings also highlight that experiencing higher-quality contraceptive care actually leads to subsequent greater satisfaction with one’s contraceptive method [14, 25]. We simultaneously find that the relationship between one’s experience of care and their subsequent contraceptive access outcomes differs based on their racial and ethnic identities. Specifically, those identifying as non-Hispanic white realize the benefit of person-centered contraceptive care on their preferred contraceptive use and satisfaction while their BIPOC-identifying peers do not.

Cross-sectional associations between patients’ experiences of contraceptive care, on one hand, and their ability to realize their contraceptive preferences with no barriers to access, on the other, are important findings that highlight the interconnectedness between these person-centered metrics. Our study, which shows a clear through line from the patient-provider encounter to patients’ subsequent reproductive autonomy, underscores the important role that health care providers play in their patients’ trajectory from care to subsequent contraceptive access. We show that positive experiences of contraceptive counseling yield person-centered positive outcomes, but potentially only for some patients. For patients identifying as BIPOC in our study, receiving high-quality contraceptive care did not necessarily lead to the same, strong association with positive outcomes that we could detect for their non-Hispanic white peers. This may indicate that there may be something different about the translation of person-centered contraceptive counseling into the realization of contraceptive preferences according to racial and ethnic identity.

At the national level, Black women report lower person-centered contraceptive counseling ratings on the specific item within the PCCC of their providers respecting them as a person compared to white women [26]. This variation of experiences in different aspects of the counseling interaction by racial identity, which respondents in our analysis mirrored, may help to explain some of the findings in our study. A wealth of literature documents that provider biases rooted in interpersonal racism, especially with regards to differential assumptions about motivations for contraceptive use or “best” methods to recommend based on (perceptions of) patients’ racial identities, can hamper individuals’ access to their preferred contraception, impeding their ability to realize full reproductive autonomy [10, 27]. Perceptions of mistrust of the broader contraceptive health care system, levels of which are higher among BIPOC individuals due, in part, to historical and ongoing experiences of discrimination and coercion within the U.S. medical system [28], are also linked to lower levels of contraceptive use [29] and preferences for less contact with a healthcare provider to access contraception [1]. To address these differential interpersonal experiences that patients in our study and others experience in the contraceptive encounter, greater emphasis on shared decision-making models that emphasize patients’ preferences and address historical inequities and medical racism is critical [30, 31]. For Black patients, especially, training all providers to address implicit bias and ensuring availability of providers of color to have more concordance between patient and provider backgrounds are two avenues for increasing positive experiences in the healthcare system [32, 33].

Beyond the clinical encounter between patients and providers, systems must be intentionally built and sustained to ensure that all people have what they need to achieve their highest level of sexual and reproductive well-being. These systems- and structural-level shifts are necessary to achieve sexual and reproductive health equity. Our study highlights that the publicly funded family planning care delivery systems in these three states may be falling short of SRHE goals when considering the role that contraceptive care delivery plays in helping people to realize their contraceptive preferences. This may especially be the case with regards to differences across patients’ racial and ethnic identities. U.S. federally supported family planning care via Title X, a federal grant program that provides dedicated funds for the delivery of sexual and reproductive healthcare and prioritizes coverage of care for patients based on their income and age, must follow established federal guidelines for delivering high-quality, equitable, and accessible care [34, 35]. Disruptions over the past several years due to changing regulations for the Title X program during the Trump administration and the COVID-19 pandemic have impeded progress in this area [36–38]. Fluctuating funding levels for the Title X system during this time period have not met the actual level of demand for restoring and building this system to fully realize its objectives [38]. Working to ensure that future funding levels for this program increase to reflect the breadth of patient and provider needs across the system, as well as ensuring that Title X regulations and the underlying statute promote comprehensive, medically sound and patient-centered care are not subject to fluctuating political support are key policy steps towards SRHE. The 2023 Black Reproductive Justice Policy Agenda, developed and endorsed by over 30 reproductive justice organizations, provides a comprehensive roadmap for embedding contraceptive equity within the larger ecosystem of reproductive justice [39]. Policy recommendations in the Agenda highlight the importance of intersectional systems- and structural-level solutions to combat both access barriers and the historical coercion of women of color within the contraceptive healthcare system, all of which are necessary strategies to move towards environments where trust between providers and their patients can be built.

Importantly, evidence indicates that people who are not linked to the health care system are likely those who experience the greatest barriers to accessing that care; our study is solely focused on those who already received contraceptive care and thus is likely not capturing those with the greatest access barriers. We hypothesize that this narrowing of the study population is a contributing factor to not identifying overall longitudinal associations with all of our access outcomes, as it is likely individuals who experience the greatest barriers to accessing care, who never make it to the family planning center, who realize the most significant consequences for subsequent contraceptive access over time. Given our focus on racial and ethnic identity in this analysis, and the large body of evidence regarding structural barriers to accessing healthcare among individuals identifying as BIPOC, focusing our stratified analyses only among those who had received recent contraceptive care likely underrepresents differences in contraceptive access across racial and ethnic identities. Future research should examine relationships between contraceptive experiences beyond the clinical context and subsequent contraceptive access outcomes to better capture perspectives of people not already linked to the contraceptive healthcare system.

While a key strength of our study is that we rely on panel data from a large sample of family planning patients, our study still has some important limitations. Key metrics of interest in our study do not change dramatically over the study period, likely because of the narrower focus on individuals who have already been linked into the healthcare system. Therefore, our ability to detect strong associations between how changes in our independent variables lead to changes in our dependent variables over time is limited. We did not explicitly power the broader RHIS clinic patient survey to examine differences in contraceptive access outcomes by race/ethnicity, so our findings stratified by racial and ethnic identity are likely a conservative representation of differentials in outcomes across these axes of identity. In addition, due to our collapsing of multiple racial and ethnic identities into one BIPOC category because of data limitations, we are unable to tease out how the findings documented in these analyses might differ across races and ethnicities. We would expect there to be differences given clear evidence that racism and coercion within the healthcare setting is differentially experienced by and perpetuated on people of color of different races [7, 8, 10, 40]. More research designed specifically to detect differences in these experiences by disaggregated racial identity is warranted.

Given evidence of substandard contraceptive care experiences and lower levels of access to care for other marginalized identities, such as queer, trans/nonbinary, and differently abled people [41, 42], there may be differentials in pathways from care to access along other intersecting axes of identities that this study was not equipped to pick up. Future research should examine how perceptions of trust and experiences of care track against each other over time, with attention to specific aspects of contact with the contraceptive healthcare system that serve as either bridges or barriers to ongoing high-quality care. Finally, two key events of relevance occurred during the study period and at differential timepoints during the fielding of surveys in each state—the 2019 Title X Final Rule and the COVID-19 global pandemic, each of which has been shown to have impacted both the delivery of family planning care as well as whether people were able to access contraception and related care during this time period. Teasing out the role of state context, and the unique or differential role that these specific events may have had on our study outcomes by state, is beyond the scope of this study.

Findings from these analyses can contribute to improved understanding of how to design equitable and person-centered systems for SRH care delivery. By highlighting the interconnectedness between patient experiences, preferences, and their subsequent access to SRH care, our findings highlight that inequities in care experiences lead to ongoing inequities in care access. Without high-quality contraceptive care, individuals are less able to realize their contraceptive preferences and overcome barriers to realizing these preferences. Addressing these inequities requires a focus on supporting providers to confront their own biases towards patients as well as building systems with a foundation of SRHE.

## References

[1] Kavanaugh ML, Zolna MR. Where Do Reproductive-Aged Women Want to Get Contraception? Journal of Women’s Health. 2023 Jun 1;32(6):657–69. doi: 10.1089/jwh.2022.0406 37099807 PMC10277980

[2] Senderowicz L, Higgins J. Reproductive Autonomy Is Nonnegotiable, Even in the Time of COVID-19. Perspectives on Sexual and Reproductive Health. 2020;52(2):81–5. doi: 10.1363/psrh.12152 32597561

[3] Holt K, Reed R, Crear-Perry J, Scott C, Wulf S, Dehlendorf C. Beyond same-day long-acting reversible contraceptive access: a person-centered framework for advancing high-quality, equitable contraceptive care. Am J Obstet Gynecol. 2020 Apr;222(4S):S878.e1-S878.e6. 10.1016/j.ajog.2019.11.1279 31809706

[4] Gavin L, Moskosky S, Carter M, Curtis K, Glass E, Godfrey E, et al. Providing quality family planning services: recommendations of CDC and the U.S. Office of Population Affairs. MMWR. 2014;63(No. RR-4):1–51. 24759690

[5] Institute of Medicine (US) Committee on Quality of Health Care in America. Crossing the Quality Chasm: A New Health System for the 21st Century [Internet]. Washington (DC): National Academies Press (US); 2001 [cited 2023 Oct 3]. Available from: http://www.ncbi.nlm.nih.gov/books/NBK222274/25057539

[6] Hart J, Crear-Perry J, Stern L. US Sexual and Reproductive Health Policy: Which Frameworks Are Needed Now, and Next Steps Forward. Am J Public Health. 2022 Jun;112(S5):S518–22. doi: 10.2105/AJPH.2022.306929 35767777 PMC10490305

[7] Kathawa CA, Arora KS. Implicit Bias in Counseling for Permanent Contraception: Historical Context and Recommendations for Counseling. Health Equity. 2020 Jul 1;4(1):326–9. doi: 10.1089/heq.2020.0025 32775942 PMC7410277

[8] Brandi K, Fuentes L. The history of tiered-effectiveness contraceptive counseling and the importance of patient-centered family planning care. Am J Obstet Gynecol. 2020;222(4S):S873–7. doi: 10.1016/j.ajog.2019.11.1271 31794724

[9] Harris LH, Wolfe T. Stratified reproduction, family planning care and the double edge of history. Curr Opin Obstet Gynecol. 2014 Dec;26(6):539–44. doi: 10.1097/GCO.0000000000000121 25379770

[10] Gomez AM, Wapman M. Under (implicit) pressure: young Black and Latina women’s perceptions of contraceptive care. Contraception. 2017 Oct;96(4):221–6. doi: 10.1016/j.contraception.2017.07.007 28756187

[11] Higgins JA, Kramer RD, Ryder KM. Provider Bias in Long-Acting Reversible Contraception (LARC) Promotion and Removal: Perceptions of Young Adult Women. Am J Public Health. 2016 Nov;106(11):1932–7. doi: 10.2105/AJPH.2016.303393 27631741 PMC5055778

[12] Frederiksen B, Ranji U, Long M, Diep K, Salganicoff A. Contraception in the United States: A Closer Look at Experiences, Preferences, and Coverage [Internet]. KFF; 2022 Nov. Available from: https://www.kff.org/womens-health-policy/report/contraception-in-the-united-states-a-closer-look-at-experiences-preferences-and-coverage/

[13] Wingo E, Sarnaik S, Michel M, Hessler D, Frederiksen B, Kavanaugh ML, et al. The status of person-centered contraceptive care in the United States: Results from a nationally representative sample. Perspectives on Sexual and Reproductive Health. 2023;55(3):129–39. doi: 10.1363/psrh.12245 37654244

[14] Kavanaugh ML, Pliskin E, Hussain R. Associations between unfulfilled contraceptive preferences due to cost and low-income patients’ access to and experiences of contraceptive care in the United States, 2015–2019. Contraception X. 2022;4:100076. doi: 10.1016/j.conx.2022.100076 35620731 PMC9126850

[15] Burke KL, Potter JE. Meeting Preferences for Specific Contraceptive Methods: An Overdue Indicator. Studies in Family Planning. 2023 Mar;54(1):281–300. doi: 10.1111/sifp.12218 36705876

[16] Kavanaugh ML, Zolna M, Pliskin E, MacFarlane K. A Prospective Cohort Study of Changes in Access to Contraceptive Care and Use Two Years after Iowa Medicaid Coverage Restrictions at Abortion-Providing Facilities Went into Effect. Popul Res Policy Rev. 2022;41:2555–83. doi: 10.1007/s11113-022-09740-4 36092460 PMC9440451

[17] Frohwirth L, Kavanaugh ML, Douglas-Hall A, MacFarlane K, Beavin C. Access to Preferred Contraceptive Strategies in Iowa: A Longitudinal Qualitative Study of Effects of Shifts in Policy and Healthcare Contexts. Journal of Health Care for the Poor and Underserved. 2022;33(3):1494–518. 36245177 10.1353/hpu.2022.0126

[18] Guttmacher. Guttmacher Institute. 2022 [cited 2022 Jan 13]. Reproductive Health Impact Study. Available from: https://www.guttmacher.org/reproductive-health-impact-study

[19] Fuentes L, Douglas-Hall A, Geddes CE, Kavanaugh ML. Primary and reproductive healthcare access and use among reproductive aged women and female family planning patients in 3 states. PLOS ONE. 2023 May 24;18(5):e0285825. doi: 10.1371/journal.pone.0285825 37224157 PMC10208491

[20] HHS.gov [Internet]. [cited 2023 Mar 8]. Code of Federal Regulations—Title 45 Public Welfare CFR 46, Informed Consent FAQs. Available from: https://www.hhs.gov/ohrp/regulations-and-policy/guidance/faq/informed-consent/index.html

[21] Zolna M, Kavanaugh M. Guttmacher Reproductive Health Impact Study Family Planning Clinic Patient Surveys [Internet]. Open Science Framework; 2023 [cited 2023 Oct 4]. Available from: https://osf.io/zbg4s/

[22] Dehlendorf C, Fox E, Silverstein IA, Hoffman A, Campora Pérez MP, Holt K, et al. Development of the Person-Centered Contraceptive Counseling scale (PCCC), a short form of the Interpersonal Quality of Family Planning care scale. Contraception. 2021 May;103(5):310–5. doi: 10.1016/j.contraception.2021.01.008 33508252

[23] Crow R, Gage H, Hampson S, Hart J, Kimber A, Storey L, et al. The measurement of satisfaction with health care: implications for practice from a systematic review of the literature. Health Technology Assessment [Internet]. 2003 Jun 26 [cited 2023 Oct 4];6(32). Available from: https://www.journalslibrary.nihr.ac.uk/hta/hta6320/10.3310/hta6320 12925269

[24] Dehlendorf C, Vittinghoff E, Silverstein I, Fox E, Logan R, Reed R, et al. Prioritizing patient experience: Validation of the person-centered contraceptive counseling measure as a performance measure. Contraception. 2023 Jul 1;123:110010. doi: 10.1016/j.contraception.2023.110010 36934954

[25] Chakraborty P, Gallo MF, Nawaz S, Smith MH, Hood RB, Chettri S, et al. Use of nonpreferred contraceptive methods among women in Ohio. Contraception. 2021 May;103(5):328–35. doi: 10.1016/j.contraception.2021.02.006 33607120 PMC10104247

[26] Welti K, Manlove J, Finocharo J, Faccio B, Kim L. Women’s experiences with person-centered family planning care: differences by sociodemographic characteristics. Contraception: X. 2022 Jan 1;4:100081. doi: 10.1016/j.conx.2022.100081 35965653 PMC9372601

[27] Dehlendorf C, Ruskin R, Grumbach K, Vittinghoff E, Bibbins-Domingo K, Schillinger D, et al. Recommendations for intrauterine contraception: a randomized trial of the effects of patients’ race/ethnicity and socioeconomic status. Am J Obstet Gynecol. 2010 Oct;203(4):319.e1–8. doi: 10.1016/j.ajog.2010.05.009 20598282 PMC3012124

[28] Roberts DE. Killing the Black Body: Race, Reproduction, and The Meaning of Liberty [Internet]. Random House/Pantheon; 1997. Available from: https://scholarship.law.upenn.edu/faculty_scholarship/2776

[29] Frost JJ, Lindberg LD, Finer LB. Young Adults’ Contraceptive Knowledge, Norms and Attitudes: Associations with Risk Of Unintended Pregnancy. Perspectives on Sexual and Reproductive Health. 2012 Jun;44(2):107–16. doi: 10.1363/4410712 22681426

[30] Dehlendorf C, Levy K, Kelley A, Grumbach K, Steinauer J. Women’s preferences for contraceptive counseling and decision making. Contraception. 2013 Aug;88(2):250–6. doi: 10.1016/j.contraception.2012.10.012 23177265 PMC4026257

[31] Dehlendorf C, Grumbach K, Schmittdiel JA, Steinauer J. Shared Decision Making in Contraceptive Counseling. Contraception. 2017 May;95(5):452–5. doi: 10.1016/j.contraception.2016.12.010 28069491 PMC5466847

[32] Treder K, White KO, Woodhams E, Pancholi R, Yinusa-Nyahkoon L. Racism and the Reproductive Health Experiences of U.S.-Born Black Women. Obstet Gynecol. 2022 Mar 1;139(3):407–16. doi: 10.1097/AOG.0000000000004675 35115434

[33] Takeshita J, Wang S, Loren AW, Mitra N, Shults J, Shin DB, et al. Association of Racial/Ethnic and Gender Concordance Between Patients and Physicians With Patient Experience Ratings. JAMA Netw Open. 2020 Nov 9;3(11):e2024583. doi: 10.1001/jamanetworkopen.2020.24583 33165609 PMC7653497

[34] Office of Population Affairs [Internet]. 2023. The Title X Family Planning Program. Available from: https://www.opa.hhs.gov/sites/default/files/2023-12/title-x-family-planning-program-2023-dec.pdf

[35] Office of Population Affairs [Internet]. 2020. Title X—Population Research and Voluntary Family Planning Programs. Available from: https://www.opa.hhs.gov/sites/default/files/2020-07/title-x-statute-attachment-a_0.pdf

[36] Office of Population Affairs. OPA Title X Family Planning Program. OASH Office of Population Affairs; 2021 Dec p. 2.

[37] VandeVusse A, Mueller J, Braccia A, Sackietey S, Frost J. Publicly Funded Clinics Providing Contraceptive Services in Four US States: The Disruptions of the “Domestic Gag Rule” and COVID-19 [Internet]. Guttmacher Institute; 2023b [cited 2023 Oct 16]. Available from: https://www.guttmacher.org/report/clinics-providing-services-in-four-us-states-disruptions-of-gag-rule-and-covid-19

[38] Hicks S. RE: Family Planning in the FY 2024 President’s Budget [Internet]. 2022 [cited 2023 Oct 16]. Available from: https://www.nationalfamilyplanning.org/file/FY24-OMB-NFPRHA-letter-final.pdf

[39] Reimagining Policy: In Pursuit of Black Reproductive Justice [Internet]. 2023 [cited 2023 Oct 16]. (2023 Black Reproductive Justice Policy Agenda). Available from: https://blackrj.org/wp-content/uploads/2023/06/RJPolicyAgenda2023.pdf

[40] Yee LM, Simon MA. Perceptions of coercion, discrimination and other negative experiences in postpartum contraceptive counseling for low-income minority women. J Health Care Poor Underserved. 2011 Nov;22(4):1387–400. doi: 10.1353/hpu.2011.0144 22080717

[41] Fletcher J, Yee H, Ong B, Roden RC. Centering disability visibility in reproductive health care: Dismantling barriers to achieve reproductive equity. Womens Health (Lond Engl). 2023 Jan 1;19:17455057231197166. doi: 10.1177/17455057231197166 37675891 PMC10486212

[42] Agénor M, Pérez AE, Wilhoit A, Almeda F, Charlton BM, Evans ML, et al. Contraceptive Care Disparities Among Sexual Orientation Identity and Racial/Ethnic Subgroups of U.S. Women: A National Probability Sample Study. Journal of Women’s Health. 2021 Oct;30(10):1406. doi: 10.1089/jwh.2020.8992 34129406 PMC8590146

